# Supplementary material for: Urinary cotinine concentration as a biomarker of environmental exposure to Nicotine in Vietnam: Results from a Nationwide Survey in 2024
Source: PLoS One. 2026 Jan 27;21(1):e0338434. doi: 10.1371/journal.pone.0338434 (PMC12843596; doi:10.1371/journal.pone.0338434)
Supplement: Attachment — Submitted filename: Response to reviwers PONE-D-25-38585_final.docx [file pone.0338434.s003.docx]

**PONE-D-25-38585**

Urinary Cotinine concentration as a biomarker of environmental exposure to Nicotine in Vietnam: Results from a Nationwide Survey in 2024

**Journal Requirements:**

1. Please ensure that your manuscript meets PLOS ONE's style requirements, including those for file naming. The PLOS ONE style templates can be found at

<https://journals.plos.org/plosone/s/file?id=wjVg/PLOSOne_formatting_sample_main_body.pdf> and

[https://journals.plos.org/plosone/s/file?id=ba62/PLOSOne_formatting_sample_title_authors_affiliations.pdf](https://track.editorialmanager.com/CL0/https:%2F%2Fjournals.plos.org%2Fplosone%2Fs%2Ffile%3Fid=ba62%2FPLOSOne_formatting_sample_title_authors_affiliations.pdf/1/010f0199944436cd-dbd83793-ae61-4678-9630-11794a9fe972-000000/krrSOtoUgsRD9G4EZnTHTNvTd_hmxzLCaRpKcps1TvI=230)

**Reply:**

**T**he authors prepared the revised manuscript following PLOS ONE's style requirements/ templates.

2. Thank you for stating the following in the Acknowledgments Section of your manuscript:
“This study was supported by the National Project “Development of a Basic Biochemical Index Set for Environmental Toxicology in the Vietnamese Working-Age Population” (Project code: ĐTĐL.CN-39/21), under the Basic Science Development Program in Chemistry, Life Sciences, Earth Sciences, and Marine Sciences (2017–2025) funded by the Ministry of Science and Technology. The authors gratefully acknowledge this valuable support”
We note that you have provided funding information that is currently declared in your Funding Statement. However, funding information should not appear in the Acknowledgments section or other areas of your manuscript. We will only publish funding information present in the Funding Statement section of the online submission form.
Please remove any funding-related text from the manuscript and let us know how you would like to update your Funding Statement. Currently, your Funding Statement reads as follows:
“SVN received the funding from the Ministry of Science and Technology as a principle investigator of the National Project “Development of a Basic Biochemical Index Set for Environmental Toxicology in the Vietnamese Working-Age Population” (Project code: ĐTĐL.CN-39/21) under the Basic Science Development Program in Chemistry, Life Sciences, Earth Sciences, and Marine Sciences (2017–2025). The funders had no role in study design, data collection and analysis, decision to publish, or preparation of the manuscript.”
Please include your amended statements within your cover letter; we will change the online submission form on your behalf.

**Reply:**

The authors revised the Acknowledgments Section (line 508-512) as follows:

“This study used the data and biospecimens collected by the National Project titled “Development of a Basic Biochemical Index Set for Environmental Toxicology in the Vietnamese Working-Age Population” which was implemented by the National Institute of Occupational and Environmental Health. The authors gratefully acknowledge this valuable support. We would like to sincerely thank the project participants”.

The authors would like to udpate the Funding Statement as follows:

“The National Project “Development of a Basic Biochemical Index Set for Environmental Toxicology in the Vietnamese Working-Age Population” (Project code: DTDL.CN-39/21) was funded by the Ministry of Science and Technology, Vietnam, under the Basic Science Development Program in Chemistry, Life Sciences, Earth Sciences, and Marine Sciences (2017–2025). The funders had no role in study design, data collection and analysis, decision to publish, or preparation of this manuscript.”

3. If the reviewer comments include a recommendation to cite specific previously published works, please review and evaluate these publications to determine whether they are relevant and should be cited. There is no requirement to cite these works unless the editor has indicated otherwise.

**Reply:**

Thank you very much for your guidance, the authors reviewed and selected appropriate references with thorough consideration.

**Reply to Reviewer: 1**

*This study presents a nationwide analysis of urinary cotinine as a biomarker for exposure to tobacco smoke (nicotine). The study itself is not novel. Surveys on tobacco smoke exposure are routinely conducted in various countries, including the USA, Brazil, Korea, and Japan. However, the introduction of a cut-point for urinary cotinine levels, adjusted for creatinine concentration, adds valuable insight to the literature.
Overall, the study is well-executed, with data processed through robust statistical analysis, yielding reliable results. Here are some suggestions for enhancing this work:*

**Reply:**

We greatly appreciate your constructive comments, and we feel that they have strengthened our manuscript. Revisions are highlighted in red in the revised manuscript. Below are our responses to the comments.

*#1. The urinary cotinine concentrations for active and passive smokers in this study should be compared with those in other global studies, and any differences should be discussed. Please review additional literature on this topic. The reviewer can suggest a few as follows, sorted by publication year:
- 2022:* [*https://doi.org/10.3390/ijerph19063746*](https://doi.org/10.3390/ijerph19063746)  *- 2021:* [*https://doi.org/10.1186/s12889-021-11265-y*](https://doi.org/10.1186/s12889-021-11265-y) *- 2021:* [*https://doi.org/10.4082/kjfm.20.0056*](https://doi.org/10.4082/kjfm.20.0056) *- 2020:* [*http://dx.doi.org/10.2174/1875318302010010060*](http://dx.doi.org/10.2174/1875318302010010060) *- 2020:* [*https://doi.org/10.21215/kjfp.2020.10.5.378*](https://doi.org/10.21215/kjfp.2020.10.5.378) *- 2019:* [*https://doi.org/10.1080/1354750X.2019.1684563*](https://doi.org/10.1080/1354750X.2019.1684563) *- 2019:* [*https://doi.org/10.1080/1354750X.2020.1797879*](https://doi.org/10.1080/1354750X.2020.1797879)

**Reply**

The authors highly appreciated your surport. We thoroughly reviewed all suggested references. However, we will provide further discussion with the results of Yang et al (2022) *(reference* [*https://doi.org/10.3390/ijerph19063746*](https://doi.org/10.3390/ijerph19063746): Exposure and Risk Assessment of Second- and Third-Hand Tobacco Smoke Using Urinary Cotinine Levels in South Korea(1)) which analyzed KNHANES VII data on 3203 Korean subjects (aged over 6 years old) during the period 2016–2018. Yang’s study used the urine cotinine cutoff of 100 μg/g-creatinine. Another reference is from Kim et al, (2019), in their literature review, the authors suggested the urine cotinine cutoffs will be approximately 33–131 μg/g-creatinine (2).

Other studies used the geometric mean of urinary cotinine concentrations or urinary cotinine levels that were different from the depedent variables of our study which were urinary cotinine to creatinine concentration/ cutoff levels. This difference makes comparison quite inappropriate.

Additional discussion was provided from line 386 to 404 under section Discussion 🡪 Optimal Cut-off Point of CCR to Classify Active Smokers and Non-Smokers.

*#2. 2. Similarly, the proposed cut-point should be compared with those suggested in other studies. In several studies, the cut-point for urinary cotinine is suggested to range from 30 to 40 µg/g-creatinine. Please review additional literature on this topic. The reviewer can suggest a few as follows, sorted by publication year:
- 2022:* [*https://doi.org/10.3390/ijerph19063746*](https://doi.org/10.3390/ijerph19063746)  *- 2020:* [*https://doi.org/10.3390/ijerph17155537*](https://doi.org/10.3390/ijerph17155537)  *- 2016:* [*https://doi.org/10.3390/ijerph13121236*](https://doi.org/10.3390/ijerph13121236)

**Reply**

The authors highly appreciated your surport and valuable suggestions. We thoroughly reviewed all suggested references.

We will add further appropriate discussions with the results of Yang et al (2022) (<https://doi.org/10.3390/ijerph19063746>) as response to the previous comments.We also add further appropriate dicussion with results with Kim et al (2016) (<https://doi.org/10.3390/ijerph13121236>) which discussed that the urinary cotinine cutoff levels were varied with different measurement methods (3). These discussions were provided from line 386 to 404 under section Discussion 🡪 Optimal Cut-off Point of CCR to Classify Active Smokers and Non-Smokers

We do not have further discussion with the results of Nishihama et al (2020) ([*https://doi.org/10.3390/ijerph17155537*](https://doi.org/10.3390/ijerph17155537)) because this study was conducted with 89,895 pregnant women who participated in the Japan Environment and Children’s Study in September 2019. In our study, we only included healthy adults who were not in their pregnancy so comparison of the two studies’ results might not be appropriate.

*#3. Surprisingly, the survey does not inquire about the types of cigarettes regularly smoked by participants. With the increasing prevalence of vaping, there may be greater nicotine exposure compared to conventional smoking. This is a significant limitation and should be discussed comprehensively.*

**Reply**

Thank you for your comment. In this study, our primary objective was to quantify tobacco smoke exposure among adults in Vietnam in 2024 using urinary cotinine to creatinine concentrations. Since nicotine from all tobacco products is metabolized into cotinine, our assessment focused on cotinine levels regardless of the specific type of tobacco exposure. We collected self-reported information on cigarette and pipe tobacco use; however, we did not distinguish between different forms of tobacco products (e.g., paper cigarettes, electronic cigarettes, etc.) in our analysis.

*#4. Please provide QA/QC information for the analysis*
**Reply**

Thank you for pointing this out. We providied additional QA/QC information in the Laboratory Analysis section (line 230-237) as follow:

Certified reference materials (CRM) ClinChek® Urine Controls, Germany level I were used for quality control the results of LC MS/MS analysis. Measured results and recoveries for cotinine in CRM ClinChek® Urine Controls, Germany are shown in the table 1. CRM has a target value of 163 µg/L, the allowable value range is from 130 µg/L to 196 µg/L.

**Table 1. Measured results and recoveries for cotinine in Certified reference materials ClinChek® Urine Controls**

| **No** | **Sample** | **Cotinine concentration (µg/L)** | **Recovery**  **(%)** | **Average cotinine concentration (µg/L)** | **Standard deviation (SD)** | **Relative standard deviation (RSD%)** |
| --- | --- | --- | --- | --- | --- | --- |
| 1 | CRM_1_ | 162.69 | 99.81 | 166.16 | 7.74 | 4.66 |
| 2 | CRM_2_ | 165.39 | 101.47 |  |  |  |
| 3 | CRM_3_ | 173.47 | 106.43 |  |  |  |
| 4 | CRM_4_ | 170.78 | 104.77 |  |  |  |
| 5 | CRM_5_ | 176.17 | 108.08 |  |  |  |
| 6 | CRM_6_ | 160.00 | 98.16 |  |  |  |
| 7 | CRM_7_ | 154.61 | 94.85 |  |  |  |

**Reply to reviewer 2:**

*#1. In your statistical analysis (lines 215, 216, and 217), please specify the exact outlier detection test you used. For example, did you use the Grubbs test (*[*https://www.graphpad.com/quickcalcs/*](https://www.graphpad.com/quickcalcs/)*) for continuous data? If so, please indicate this clearly and, if desired, provide the reference.*

**Reply**

Thank you for your constructive comment regarding data variability. To ensure robust interpretation of cotinine-creatinine corrected concentrations, we conducted a comprehensive outlier assessment using multiple statistical approaches (IQR rule, z-score threshold, and percentile method), along with a biological plausibility review. Values flagged by at least two methods were classified as outliers (32 participants; 2.97%). Instead of excluding them directly, we retained these observations but marked them with an indicator variable to support sensitivity analyses and avoid bias introduced by data removal. Additional explanation was provided in the Statistical Analysis (line 255-262) as follow:

*“Cotinine concentrations normalized by urinary creatinine were evaluated for extreme values using multiple statistical approaches, including the interquartile range (IQR) rule, z-scores (> |3|), and the 99th percentile threshold. A biological plausibility check was also performed based on published reference ranges for urinary cotinine in smokers. Data points that were identified as extreme by at least two independent methods were classified as outliers. Using this combined approach, 32 outliers (2.97% of total samples) were flagged. These values were retained in the dataset with an indicator variable for sensitivity analyses and model robustness checks.”*

*#2. At the end of line 236, ensure you include the confidence interval used (e.g., 95%), as this information is essential.*

**Reply**

Thank you for pointing this out. The authors indicated the use of odd ratios and 95% confidence interval in the Statistical Analysis section line 276.

*#3. For Figure 1, please indicate the sensitivity and specificity test values, alongside the CCR cut-off value, within the figure or its legend, as the cut-off selection in the ROC curve depends on these metrics.*

**Reply**

Thank you for your comments, the authors included the sensitivity and specificity test values, CCR cut-off values and AUC in the Figure 1 in line 311.

**Reply to reviewer 3:**

*This manuscript describes the first nation-wide study to monitor smoking prevalence and exposure to second hand smoke in Vietnam. So far, most studies in Vietnam and developing countries only used questionnaires to record and estimate prevalence, thus the data are of great interest to public health agencies and could serve as evidence for future tobacco control policies.*

**Reply:**

We greatly appreciate your constructive comments, and we feel that they have strengthened our manuscript. Revisions are highlighted in red in the revised manuscript. Below are our responses to the comments.

*The authors have presented all the data, with unexpected findings such as lower smoking prevalence in farmers and lower income earners than official and high income earners.*

*However, I suggest the authors to consider some issues below:*

*#1. The recruitment process has to be described in detail to show that the sampled populations are representative.*

**Reply**

Thank you for pointing this out. The authors provided details on the sampling method in the Study Design and Population section line 153 to 176 as follow:

*“Study participants were selected via multi-stage sampling procedure as follow:*

*Stage 1. Selecting provinces: To ensure that the research sample represents the Vietnamese working-age population in different parts of Vietnam, we randomly selected one province from each of seven administrative and socio-ecological regions of the country. The selected provinces included Lang Son province (Northeast region), Phu Tho province (old Hoa Binh, Northwest region), Hanoi city (Red River Delta region), Nghe An province (North Central region), Khanh Hoa province (South Central Coast and Central Highlands region). Ho Chi Minh city (Southeast region), Can Tho city (Mekong Delta region).*

*Stage 2. Selecting commune/ward areas: In each province/city, we randomly selected one district/county. In each district/county, we intentionally selected one ward/commune that were not located near mining, metallurgy, pesticide production, large industrial zones, had no craft villages with the risk of exposure to toxic chemicals, and no cultivation of industrial crops. There were seven ward/communes were invited to join the study.*

*Phase 3. Selecting subjects. Local health workers under the guidance of study staff prepared a list of subjects who met the study inclusion criteria. Subjects were stratified by sex (approximately 1:1 male-to-female ratio) and across four age groups (18–24, 25–34, 35–44, 45–60 years). From the above list, 20 subjects were randomly selected in each age group with equal sex ratio. Thus, 160 subjects (80 men and 80 women) were invited to join the study in each commune/ward. During the data collection process, if the subject refused to join or end their participation, another study subject would be randomly selected from the initial list. In total, 1077 healthy Vietnamese people in working age participated in the study”*

*#2. The justification of spot urine is not correct in my opinion. First void samples will probably give more consistency and be complemented by the questionnaires about how many cigarettes you smoked yesterday*

**Reply**

Thank you for your valuable comments. We agree that collecting first-void morning urine samples is ideal for assessing cotinine levels. However, due to the design of our community-based study, urine samples were collected onsite only when participants visited the research site. This approach was necessary because we also analyzed the samples for metals and other metabolites, and therefore could not ensure proper cleanliness and preservation if sampling materials were distributed in advance. To address potential variability in urine dilution, we normalized cotinine results using urinary creatinine concentrations. We also include this as one of other limitations of our study at the end of the discusison section (line 488-495)

*#3. I am not an expert in statistics but I wonder whether the unexpected findings such as the low prevalence in farmers and high prevalence in Ho Chi Minh city have anything to do with the power of the data (i.e. the sample size at provincial level is small and thus prone to high uncertainty).*

**Reply**

The unexpectedly low prevalence among farmers and the higher prevalence in Ho Chi Minh City may be explained by geographical variability in smoking behavior, as well as differences in accessibility and affordability of tobacco products. For example, despite being the second largest city in Vietnam, Hanoi demonstrated the lowest prevalence of CCR-based smokers in our study. These variations likely reflect differences in lifestyle, levels of environmental tobacco smoke exposure, and the enforcement of smoke-free policies across provinces and cities.

The lower prevalence observed among farmers may be related to the increasing price of tobacco products and reduced affordability in rural areas. Additionally, our findings indicate that higher-income groups have greater odds of being classified as smokers, further supporting the role of economic factors in shaping tobacco use patterns.

The primary objectives of our study were to: (i) determine the optimal urine cotinine cut-off that discriminates smokers from non-smokers; (ii) estimate the misclassification rate between self-reported smoking and urinary cotinine; and (iii) explore the distribution of tobacco smoke exposure among Vietnamese adults using urinary cotinine concentration. Therefore, we did not prepare an in-deep discussion on specific CCR-based smoking prevalence figures—such as by location, occupation, gender—as this was not a central focus of our analysis.

*#4. The data about female smoking prevalence is also interesting as people usually assume very low level of smoking in female in Asia in general. Question to the authors is that whether the relatively high prevalence in female is due to the “false positive” diagnosis using the CCR of 21 (I don’t think you need to be super accurate with 3 significant figures).*

**Reply**

Thank you for your thoughtful comment. CCR reflects total environmental nicotine exposure, including active smoking as well as passive exposure from second-hand (SHS) and third-hand smoke (THS). According to WHO, although the proportion of women who actively smoke in Viet Nam is low, 48% are regularly exposed to SHS at home and 25% are exposed in the workplace (4). Such widespread exposure may help explain our finding of a relatively high prevalence of CCR-based active smokers among females. In contrast, smoking prevalence in women reported in previous studies has been based primarily on self-reported data, which may underestimate smoking behavior due to social desirability bias.

The primary objectives of our study were to: (i) determine the optimal urine cotinine cut-off that discriminates smokers from non-smokers; (ii) estimate the misclassification rate between self-reported smoking and urinary cotinine; and (iii) explore the distribution of tobacco smoke exposure among Vietnamese adults using urinary cotinine concentration. Therefore, we did not prepare an in-deep discussion on specific CCR-based smoking prevalence figures—such as by location, occupation, gender—as this was not a central focus of our analysis.

*#5. Similarly, the discussion about under-reporting of smoking status, my layman understanding is that the research team, not only of this study but all studies, would have had to declare in the Participant Information Sheet that they would analyse the urine samples for tobacco biomarkers. Therefore, it would be a real surprise if the participants still tried to hide their smoking status in the questionnaires. To me, it may well be the effect of the artificial CCR values.*

**Reply**

Thank you for pointing this out. The author added this description at the end of the Introduction to clarify that this study was a part of the national project, in which, a variety of chemical exposures were measured, including nicotin exposure (via cotinine levels) (line 137-140).

*“This study was a part of the National Project titled “Development of a Basic Biochemical Index Set for Environmental Toxicology in the Vietnamese Working-Age Population” in 2025 under the management of the National Institute of Occupational and Environmental Heatlh.”*

Our study complied with the Ethical requirements of the project, we sent Information sheet to study participants to seek their consent for the collection of their biosamples (including urine) to measure various biomarkers (not only cotinine).

We also revised the wordings in the section titile “Misclassification between Self-Reported Exposure and Optimal CCR” in line 406-407 as follow:

*“Despite the overall correlation between CCR and self-reported exposure, our findings highlight the discordance on smoking status, particularly among smokers.”*

*#6. If possible, try to present the data in figures rather than table form.*

**Reply**

Thank you for pointing this out. We transformed table 2 (Distribution of smoking exposure statuses by CCR cut-off value of 20.947 µg/g) into figure 2 (line 317). We would like to keep other three tables to provide the comprehensive data more effectively.

*#7. If possible, please compile a range of CCR values from different studies*

**Reply**

Thank you for pointing this out. The authors provided more discussion and comparison with CCR values from other studies in the section “Optimal Cut-off Point of CCR to Classify Active Smokers and Non-Smokers” from line 386 to 404 as follow:

*“This cut-off value is consistent with findings from other population-based studies in Asia and Europe, which have proposed CCR thresholds ranging from 15 to 30 µg/g creatinine depending on demographic and environmental factors (5-9). However, our estimated threshold (20.947 µg/g) is notably lower than the value reported by Yang et al. (2022), who analyzed KNHANES VII data from 3,203 Korean participants aged ≥6 years and used a urine cotinine cut-off of 100 µg/g creatinine (1). It is also lower than the range suggested in Kim’s literature review (2019), which reported cut-offs between 33 and 131 µg/g creatinine (2). These variations across studies may reflect differences in race and ethnicity, smoking behavior, environmental tobacco smoke exposure, and the implementation of smoke-free policies. In addition, the urinary cotinine cutoff levels were also varied with different measurement methods (3).*

*Nevertheless, our cut-off value represents a reliable and context-specific biomarker threshold for accurately classifying tobacco exposure in Vietnam, where underreporting of smoking remains a concern. Importantly, CCR offers a key advantage over self-reported data, as it provides an objective measure of exposure unaffected by social desirability bias or recall limitations—issues commonly encountered in tobacco research (9-12). This objective measurement is particularly valuable for evaluating the effectiveness of smoke-free policies and for identifying populations at high risk of tobacco exposure.”*

We limited our references to studies conducted among healthy adults that measured cotinine normalized to creatinine, ensuring comparability with our study findings.

**Relevant references to this Response to reviewers.**

1.	Yang J, Hashemi S, Han W, Song Y, Lim Y. Exposure and Risk Assessment of Second- and Third-Hand Tobacco Smoke Using Urinary Cotinine Levels in South Korea. International Journal of Environmental Research and Public Health [Internet]. 2022; 19(6).

2.	Kim S. Chapter 51 - Overview of Cotinine Cutoff Values for Smoking Status Classification. In: Preedy VR, editor. Neuroscience of Nicotine: Academic Press; 2019. p. 419-31.

3.	Kim S. Overview of Cotinine Cutoff Values for Smoking Status Classification. Int J Environ Res Public Health. 2016;13(12).

4.	World Health O. Women and Tobacco in Viet Nam: The Hidden Threat 2023 [Available from: <https://www.who.int/vietnam/news/commentaries/detail/women-and-tobacco-in-viet-nam--the-hidden-threat>.

5.	Heo HC, Byun YS, Sohn SH, Jo SM, Park SK, Sakong J. Validity assessment of self-reported smoking status in firefighters using the urine cotinine test. Ann Occup Environ Med. 2020;32(0):e2.

6.	Abdullah N, Jamal R, loi m, Chin S-F, azlina e, Syed Zakaria SZ. Validation of self-reported smokers and second hand smoker exposure by urine cotinine: The Malaysian Cohort Project. Journal of Biomedicine and Translational Research. 2019;5.

7.	Tranfo G, Pigini D, Paci E, Marini F, Bonanni RC. Association of exposure to benzene and smoking with oxidative damage to nucleic acids by means of biological monitoring of general population volunteers. Environ Sci Pollut Res Int. 2017;24(16):13885-94.

8.	Kim KY, Myong JP, Kim HR, Lee HE, Jang TW, Koo JW, et al. Dose-related effect of urinary cotinine levels on pulmonary function among Korean women. The international journal of tuberculosis and lung disease : the official journal of the International Union against Tuberculosis and Lung Disease. 2014;18(5):622-7.

9.	Paci E, Pigini D, Bauleo L, Ancona C, Forastiere F, Tranfo G. Urinary Cotinine Concentration and Self-Reported Smoking Status in 1075 Subjects Living in Central Italy. Int J Environ Res Public Health. 2018;15(4).

10.	Boykan R, Messina CR, Chateau G, Eliscu A, Tolentino J, Goniewicz ML. Self-Reported Use of Tobacco, E-cigarettes, and Marijuana Versus Urinary Biomarkers. Pediatrics. 2019;143(5).

11.	Meadows AL, Guenthner G, Broster LS, Luo AY, Martin CA. Assessing adolescent tobacco use in a substance use treatment program with self-report and biological measures. Addict Behav. 2020;107:106424.

12.	Wang R, Hall JM, Salloum RG, Kates F, Cogle CR, Bruijnzeel AW, et al. Prevalence of Underreported Nicotine Exposure Among US Nonsmoking Adults: A Comparison of Self-Reported Exposure and Serum Cotinine Levels From NHANES 2013-2020. Nicotine Tob Res. 2024;26(3):298-306.
